# REMA: A Rich Elastic Mixed Attention Module for Single Image Super-Resolution

**DOI:** 10.3390/s24134145

**Published:** 2024-06-26

**Authors:** Xinjia Gu, Yimin Chen, Weiqin Tong

**Affiliations:** 1 School of Computer Engineering and Science, Shanghai University, Shanghai 200444, China; 2 School of Information, Shanghai Jian Qiao University, Shanghai 201306, China

**Keywords:** single-image super-resolution, Rich Structure, attention, rich elastic mixed attention

## Abstract

Detail preservation is a major challenge for single image super-resolution (SISR). Many deep learning-based SISR methods focus on lightweight network design, but these may fall short in real-world scenarios where performance is prioritized over network size. To address these problems, we propose a novel plug-and-play attention module, rich elastic mixed attention (REMA), for SISR. REMA comprises the rich spatial attention module (RSAM) and the rich channel attention module (RCAM), both built on Rich Structure. Based on the results of our research on the module’s structure, size, performance, and compatibility, Rich Structure is proposed to enhance REMA’s adaptability to varying input complexities and task requirements. RSAM learns the mutual dependencies of multiple LR-HR pairs and multi-scale features, while RCAM accentuates key features through interactive learning, effectively addressing detail loss. Extensive experiments demonstrate that REMA significantly improves performance and compatibility in SR networks compared to other attention modules. The REMA-based SR network (REMA-SRNet) outperforms comparative algorithms in both visual effects and objective evaluation quality. Additionally, we find that module compatibility correlates with cardinality and in-branch feature bandwidth, and that networks with high effective parameter counts exhibit enhanced robustness across various datasets and scale factors in SISR.

## 1. Introduction

Single image super-resolution (SISR) aims to rebuild a high-resolution (HR) image based on its low-resolution (LR) counterpart. It is widely used in digital multimedia, facial recognition, remote sensing image restoration, medical image processing, and other domains [1], and many SISR algorithms have been proposed, including interpolation, reconstruction, algebraic characteristics, and learning-based methods [2,3]. In recent years, there have been remarkable advancements in deep learning-based SISR algorithms. However, one of the major challenges of deep learning-based algorithms is high-frequency detail preservation. Numerous studies have proposed diverse algorithms to address this challenge, including residual learning [4,5], recursive structures [6,7,8], dense connections [9,10,11], and multi-path learning [12,13]. In recent times, attention-based algorithms have gained prominence, notably after the popularity of Transformer-based algorithms. In fact, there have already been plenty of studies proposing attention-based SISR methods [14,15,16,17] to restore details. Most studies prefer to design a specific SISR network utilizing attention rather than a plug-and-play attention module to improve the reconstruction quality, resulting in a lack of flexibility in methods. And, only a few researchers have proposed flexible attention modules for SR tasks [18,19,20], except for directly plugging classic attention modules into SR networks [21,22].

In fact, many researchers have solely focused on proposing size-oriented attention modules to enhance performance without increasing or even reducing model complexity. However, in real-world scenarios, there is a significant number of tasks that prioritize performance over size, rather than solely emphasizing low-complexity modules with limited performance improvement. Therefore, to address the requirements of various tasks effectively, a flexible module should encompass both size-oriented and performance-oriented characteristics, which are aspects that are rarely discussed. Moreover, according to our experiment results, some size-oriented modules may function effectively within one network; however, their compatibility with other networks may not be guaranteed. Indeed, this raises a more general question in deep learning: why does a plug-and-play module work in one network but not in another? And what are the factors influencing the performance of a plug-and-play module? These issues are not well-studied.

To address the challenges mentioned above, we identify the influential factors affecting the performance of a plug-and-play module and propose the rich elastic attention module (REMA), which is a plug-and-play attention module for SISR. For the flexibility of the module, we propose Rich Structure, which allows seamless switching between size-oriented and performance-oriented modes to accommodate various requirements and ensure compatibility with different networks. And Rich Structure is the basic structure of REMA.

From the attention module’s perspective, it is essential to identify the key features affecting SR quality. Thus, we divide SISR into two steps: (1) upsampling LR images to the target size; and (2) minimizing the difference between the resized image and the ground-truth image, succinctly referred to as ‘upscaling’ and ‘denoising’. An effective attention module should highlight key features throughout this process. Building upon the structure and inspiration from CBAM [23], REMA enhances key feature representation in these steps from spatial and channel aspects by enriching the in-module feature pass-through. Using the proposed Rich Structure, REMA can seamlessly switch between size-oriented and performance-oriented modes, ensuring flexibility for different requirements by controlling the bandwidth of in-module features pass-through.

To evaluate the effectiveness of REMA, we integrate it into our proposed modified EDSR [4] and name the resulting model REMA-SRNet. Extensive experiments are conducted on commonly used SR benchmarks. We compare REMA with other comparative algorithms and plug-and-play attention modules. The results demonstrate the effectiveness of Rich Structure, REMA, and REMA-SRNet.

In summary, the main contributions of this paper are as follows:

- We identify the key factors affecting the performance of a plug-and-play module and propose Rich Structure, enabling seamless switching between size-oriented and performance-oriented modes for a plug-and-play module to satisfy the diverse needs of different tasks.
- We propose a SISR attention module, based on Rich Structure, called REMA, consisting of RSAM and RCAM. RSAM employs a creative method to enhance performance through learning LR-HR mapping mode and multi-scale feature fusion. RCAM enhances the overall performance by learning and reducing noise caused by upsample operations and dimension–resolution changes led by convolution operations, using interactive learning. REMA can be easily integrated into networks with various architectures and significantly improve detail reconstruction accuracy at different scale factors.
- Extensive experiments demonstrate that REMA can carry a simple ResNet backbone SR network to the state of the art while balancing performance and model size. Moreover, the impact of the number of parameters on a module’s effectiveness and the overall networks’ robustness across different datasets and scale factors is comprehensively discussed in the experiments.

The remainder of this paper is organized as follows: Section 2 provides a brief overview of related work on deep learning-based SISR networks, attention modules, and attention-based SR models. In Section 3, we detail our proposed REMA, including problem analysis, overall structural design, and module architecture. Section 4 validates the effectiveness of our method, compares its performance with existing alternatives, and highlights its significant advantages. Finally, Section 5 summarizes the study and outlines directions for future work.

## 2. Related Works

### 2.1. Deep Learning-Based SR Methods

SRCNN is the first CNN-based end-to-end SISR network [24]. It interpolates the input image to the target size and employs three convolution layers for LR-HR non-linear mapping learning. SRCNN preserves more details than traditional methods, leading to its widespread adoption. Subsequently, CNN-based SISR methods have gained popularity. Examples include ESPCN [25] and FSRCNN [26], which directly take LR images as inputs directly to reduce complexity and increase network speed. ESPCN uses sub-pixel convolutional layers as reconstruction layers, while FSRCNN employs deconvolution layers for HR reconstruction.

To enhance performance, many researchers have integrated techniques such as residual learning, dense connections, recursive structures, and multi-scale or multi-level fusion into their networks. For instance, Kim et al. proposed VDSR [5], which makes the network deeper through residual learning and gradient clipping to improve reconstruction quality. EDSR [4] employs more residual blocks without batch normalization layers to deepen the network and utilizes pixel shuffle to optimize reconstruction performance. Methods like DRRN [7] and DRCN [6] introduce recursive structures to share parameters among layers and deepen the network without significantly increasing the model size. Others, such as RCAN [27], implement a cascading mechanism on a residual network to reuse hierarchical features and balance the number of parameters and accuracy.

Additionally, MSRN [28] creates two sub-branches and uses convolutions of different sizes in a residual block, fusing features interactively to obtain multi-scale features. The multi-scale dense convolutional network (MDCN) [9] densely connects each layer in multi-scale residual blocks to fully utilize multi-scale features within the block. Moreover, ESRGCNN [29] adapts group convolutional residual blocks for multi-level feature fusion and computational cost reduction. UNetSR [30] directly realizes shallow–deep feature fusion via skip connections, akin to U-Net architecture.

According to these studies, dense connections, recursive learning, multi-scale or multi-level feature fusion, and other techniques share a common goal. They aim to efficiently create and learn features at different scales within the backbone structure, a critical aspect of improving CNN-based SISR algorithms.

### 2.2. Attention and Attention-Based SR Models

Attention is a method used to recalibrate the weights of input features in deep learning, aiding models in focusing on key features. In fact, attention-based modules find wide application in various computer vision tasks. The squeeze-and-excitation (SE) block [31] was introduced to adjust informative features within channels. Woo et al. [23] proposed the convolutional block attention module (CBAM), incorporating both channel and spatial attention to adjust feature weights. Coordinate attention (CA) [32] embeds positional information into channel attention, facilitating the capture of long-range dependencies while preserving precise positional information.

Attention-based methods are also prevalent in SISR tasks. RCAN [27] implements a residual-in-residual (RIR) structure with channel attention, enhancing performance by fusing high- and low-frequency features via skip connections. DRLN [10] combines densely connected layers with residual blocks and incorporates a Laplacian pyramid attention mechanism to enhance image quality. Multi-scale feature fusion block (MSFFB) utilized in a multi-scale channel and spatial attention module (CSAM) in MCSN [33] facilitates multi-scale feature representation learning, enhancing the feature selection ability of the channel attention module. PAN [18] employs a pixel attention module in the backbone and upscale layers, generating a 3D attention map at the pixel level to improve performance with fewer parameters. PRRN [34] incorporates a progressive representation recalibration block to extract meaningful features by utilizing pixel and channel information and employing a shallow channel attention mechanism for efficient channel importance learning. RNAN [35] proposes residual non-local attention to obtaining non-local hybrid attention, further enhancing performance by adaptively adjusting the interdependence between feature channels. Dynamic attention, as used in attention to network (A2N) [36], comprises non-attention component branches and composite attention branches to dynamically suppress unnecessary attention adjustments. The non-local spatial attention network (NLSN) [20] optimizes the computational cost of non-local attention via sparse attention. SwinIR [16] and Swin2SR [17] construct networks based on the Vision Transformer, achieving superior performance.

Few studies focus on plug-and-play attention modules for SISR tasks. Wang et al. [19] proposed the lightweight attention module BAM to suppress large-scale feature edge noise while retaining high-frequency features, which is the most relevant research to our topic. BAM includes the adaptive context attention module (ACAM) for noise reduction and the multi-scale spatial attention module (MSAM) for preserving high-frequency details.

## 3. Methodology

### 3.1. Motivation and the Overall Framework

In our proposed module, the objective is to cater to the requirements of both performance-prioritized and size-prioritized tasks. Therefore, the initial focus is on maximizing performance to meet the demands of performance-prioritized tasks. Subsequently, efforts are directed toward controlling the module size to align with the needs of size-prioritized tasks. Consequently, all parameter-friendly designs are not considered during the initial stage of the module design process. This concept permeates throughout the entire module design, distinguishing our approach from others that opt for lightweight structures directly in their methods. However, this does not mean module size is not important at all for us. Indeed, this is a problem with parameter efficiency. A parameter-efficient module should not only use fewer parameters to exchange limited performance improvement but also boost the performance with more parameters and reach parameter efficiency globally. And ‘Rich Structure’ is proposed for this purpose. Table 1 illustrates the implications of nouns, abbreviations, and symbols used in the following text.

### 3.2. Module with Rich Structure

For a module, the flexibility involves more than just being plug-and-play, it also involves robustness across different datasets and compatibility to networks with varying characteristics. Identifying influential factors related to these aspects is crucial. Our experiments reveal that key factors affecting the plug-and-play module performance include the overall shape (cardinality, channel bandwidth, and depth) and task-specific effective algorithms. Hence, we propose REMA based on these considerations.

Current plug-and-play attention modules can be categorized into two types based on cardinality (the number of branches with feature transformation): single-branch modules like CBAM, SE, and PA, and multi-branch modules like CA and BAM. However, our experiments show that single-branch modules, which we define as having a plain structure, exhibit less performance improvement than most multi-branch modules when facing input features with higher complexity. Thus, our method is designed as a multi-branch structure to ensure compatibility.

**Figure 1:**
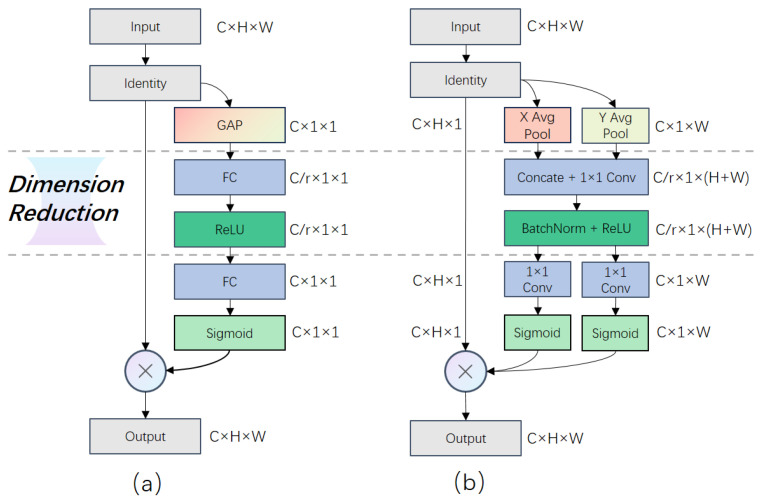
Illustration of dimension reduction in size-oriented attention modules. (**a**) Dimension reduction in SE-like modules, and the channel attention module of BAM and CBAM and their variants. (**b**) Dimension reduction in CA.

**Figure 2:**
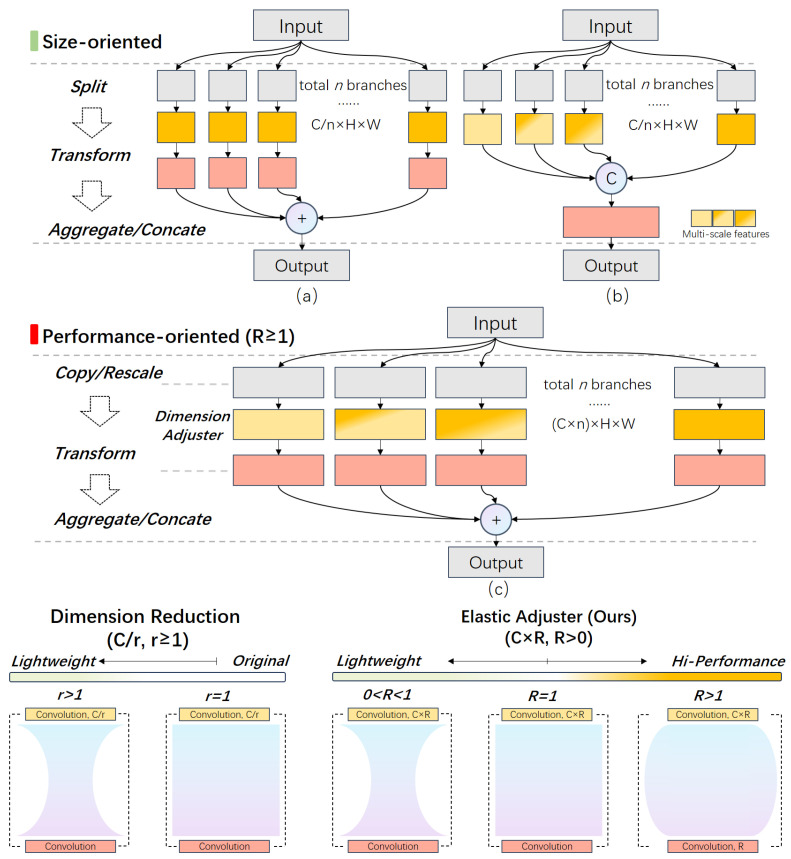
Illustration of size-oriented and performance-oriented modules. (**a**) ResNeXt-like block. (**b**) Inception-like block. (**c**) Module with Rich Structure (ours).

Attention modules with multiple branches, such as Inception-like [37] and ResNeXt [38], or Res2Net-like blocks [39], may encounter challenges related to size-oriented designs, leading to reduced robustness and overall performance across various scale factors in the SISR task. These modules adopt a similar approach to parameter control. For instance, prevalent Inception-like modules split the input feature maps along the channel dimension, transform the features, and then concatenate them for fusion. Likewise, ResNeXt and Res2Net employ bottleneck or grouped convolution to split, transform, and aggregate or concatenate features in the final stage. They all follow a ’split–transform–aggregate or concatenate’ structure to balance performance and module size, utilizing the bottleneck structure to split input features. Additionally, single-branch attention modules utilize this structure to adjust their size. Figure 1 and Figure 2 illustrate how these methods split features or control module sizes using the dimension reduction ratio (r). In other words, the ‘bottleneck’ structure can become a performance bottleneck under certain conditions.

However, the issue does not lie solely with the bottleneck structure. In fact, the real concern that deserves more attention is why the focus remains solely on the reduction in dimensions, or in other words, why finding a ratio to minimize the model size while maintaining performance is the predominant research direction. What would occur if a similar bottleneck structure were employed but with increased dimensions, i.e., widening the bandwidth of channels for feature pass-through, rather than reducing it? Only a few studies have addressed this question, such as [40,41]; the authors approached the topic from the perspective of the entire backbone, comparing the widened residual and Inception block with a deeper backbone, demonstrating the effectiveness of widening the bandwidth of channels. Our experiments also prove this from the module perspective. In other words, switching between size-oriented and performance-oriented modules could be unified within the same framework.

Therefore, Rich Structure is proposed as a multi-branch structure with a bi-directionally adjustable channel bandwidth of features in each branch (Figure 2). Specifically, in our proposed method, instead of using ‘split–transform–concatenate/aggregate structure’, we directly copy or rescale the inputs to different scales, transform features in each branch, and then aggregate them together. In other words, the structure is ‘copy/rescale–transform–aggregate’. Therefore, the overall width of the features in our module will be much larger and appear fatter, thus denoted as the Rich Structure. On the other hand, dimension reduction (C/r,r∈[1,+∞)) is replaced with the proposed elastic adjuster (C×R,R∈(0,+∞)). When R∈(0,1), the module functions akin to a ‘split–transform–concatenate/aggregate’ structure to fulfill the requirements of size-prioritized tasks. Conversely, when R∈[1,+∞), the module utilizes additional parameters to enhance performance. Thus, with the help of Rich Structure, REMA could seamlessly switch between size-oriented and performance-oriented modes to ensure flexibility to different requirements.

### 3.3. Rich Elastic Mixed Attention (REMA)

As mentioned above, the performance-related factors include the shape of the module and task-specific effective algorithms. For the former, we design Rich Structure to ensure compatibility with inputs of varying complexity and flexibility for different tasks. However, it is far less important than the latter. Thus, RSAM and RCAM are designed based on the characteristics of SISR, and Rich Structure amplifies their effectiveness. RSAM and RCAM function like miniature SR networks in REMA.

The goal of deep learning-based SISR tasks is to minimize the difference between the reconstructed image and the real HR image, which can be expressed by the following formula [42]:

(1) $${\hat{\theta }}_{F}=arg_{\theta_{F}}minL\left(I_{SR},I_{y}\right)+\lambda \Phi \left(\theta \right)$$

where $\theta_{F}$ denotes the parameters of the SR model *F*. *L* devotes the loss between the reconstructed image $I_{SR}$ and the ground-truth HR image $I_{y}$, and ${\hat{\theta }}_{F}$ denotes the model parameter that minimizes *L*. $\Phi \left(\theta \right)$ is the regularization term, and λ serves as the trade-off parameter employed to adjust the proportion of the regularization term. In other words, the purpose of deep learning-based SISR models is to find the ${\hat{\theta }}_{F}$ to make $I_{SR}$ as close to $I_{y}$ as possible.

From the module perspective, the key is to identify features that deserve more attention during the process mentioned above. To simplify the problem, we decompose the HR reconstruction process into two steps: upscaling the LR image to the target size and eliminating the difference in details between the upscaled image and the real HR image. The process can be expressed in the following formula:

(2) $$I_{y}=f\left(I_{LR}⊗M_{up},D_{HR}\right)$$

where $I_{LR}$ refers to the low-resolution image, $M_{up}$ is the LR-HR upscale mapping mode. And $D_{HR}$ denotes the difference between the upscaled LR image $I_{LR}⊗M_{up}$ and $I_{y}$.

**Figure 3:**
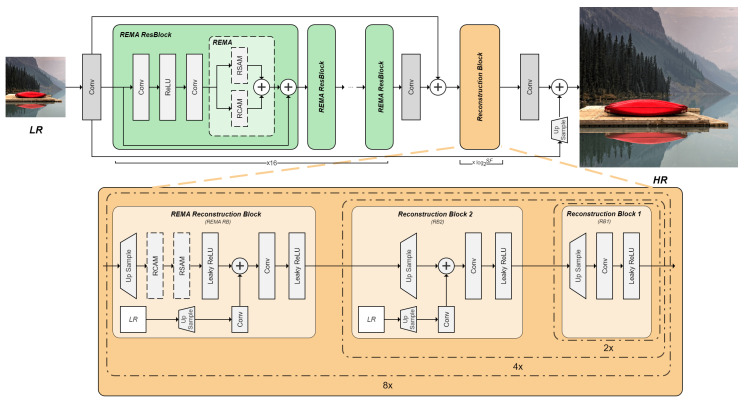
Illustration of the proposed REMA-SRNet. The backbone of REMA-SRNet is based on residual blocks incorporating REMA (REMA ResBlock). The reconstruction layers utilize bilinear upsampling followed by a 3 × 3 convolution and Leaky ReLU layers. REMA is applied at 4× and 8× upscaling, with a long skip connection from the bilinear-upscaled LR input, followed by a 1 × 1 convolution for dimension alignment. SF denotes the scale factor. For 2×, 4×, and 8× reconstruction, the number of REMA ResBlocks is 16 and the number of reconstruction blocks is 1, 2, and 3, respectively.

Obviously, the key to high-quality HR image reconstruction lies in the accurate estimation of $M_{up}$ and $D_{HR}$. Therefore, inspired by CBAM, which enhances feature representation from both spatial and channel aspects, we propose a rich spatial attention module (RSAM) and a rich channel attention module (RCAM) to improve SISR network performance. Unlike CBAM, we eschew lightweight design and instead increase cardinality, the in-branch channel dimensions, and depth. Specifically for SISR tasks, the inadequacy of CBAM and other lightweight attention modules results in a lack of sufficient space for feature maps with various resolutions for interactive learning, which is crucial for $M_{up}$ and $D_{HR}$ estimation. Since learning LR-HR mapping involves avoiding details missing due to resolution changes, there should be at least one pair of feature maps with different resolutions. Therefore, a multi-branch structure is employed in both RSAM and RCAM to enrich the in-module features passed through to aid SISR networks in learning $M_{up}$ and $D_{HR}$. On the other hand, a multi-branch structure also ensures better multi-scale and multi-level feature fusion for enhancing long-range dependency learning [43], which has already been proven effective in other studies. Thus, we combine multi-scale fusion, LR-HR interactive learning, and attention mechanisms to propose REMA. To verify the effectiveness of REMA for SISR tasks, we apply REMA to a simple ResBlock-based backbone SISR network named REMA-SRNet and compare it with other methods. We apply RSAM and RCAM in parallel at the ResBlock to enhance the backbone performance. Additionally, we fuse features from the LR image and integrate REMA into the reconstruction block to improve performance under high-scale factors. The detailed structures of REMA and REMA-SRNet are illustrated in Figure 3.

### 3.4. Rich Spatial Attention Module (RSAM)

**Figure 4:**
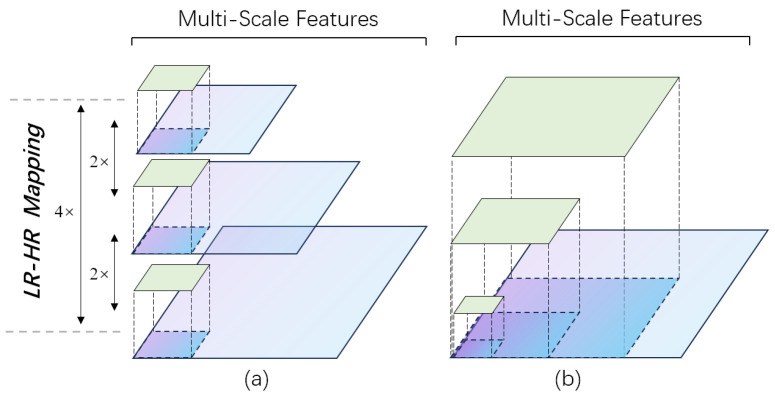
The difference in multi-scale feature generation between RSAM and other conventional methods (assuming the scale factor is 2×). (**a**) RSAM (ours) learns multi-scale features and LR-HR mapping together. (**b**) Conventional methods (like ASPP and Inception blocks) can only obtain multi-scale features.

RSAM aims to enhance long-range feature extraction and non-linear LR-HR mapping mode $M_{up}$ learning through dynamic multi-scale feature fusion with spatial attention. The main difference between RSAM and other widely used multi-scale feature fusion methods lies in the construction of the feature pyramid. As shown in Figure 4, in contrast to methods that utilize convolutions with different kernel sizes [44], or lightweight convolutions like dilated or factorized convolution [45] to learn and fuse features from the same feature maps, RSAM constructs the feature pyramid from rescaled input feature maps based on the scale factor.

Thus, regardless of how the scale factor changes, RSAM could learn $M_{up}$ correctly. Within this module, RSAM generates three LR-HR pairs and scans them with receptive fields of the same size to fuse features. Obviously, the advantage of our method is that such a design can obtain multi-scale features as well as preserve the complete structural information of LR-HR mapping, which is key to SISR tasks.

**Figure 5:**
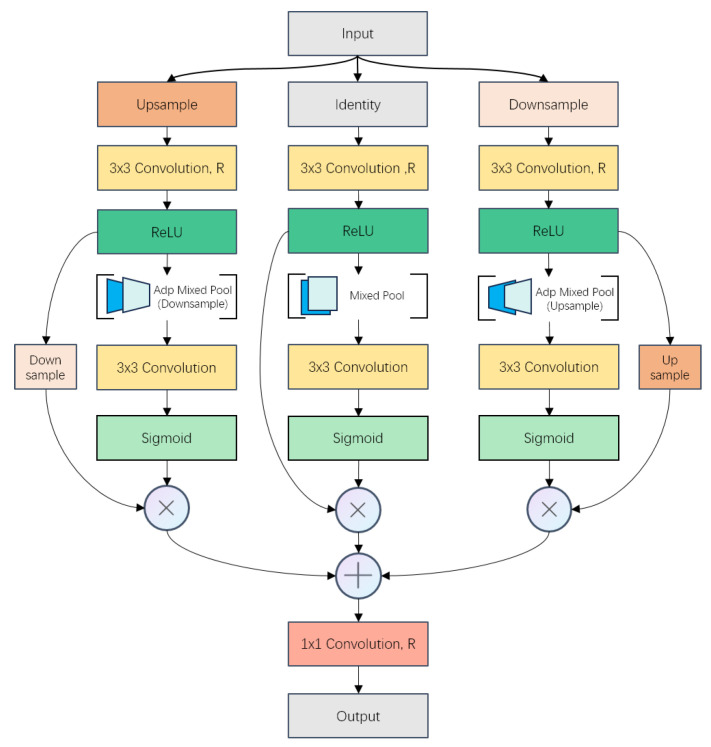
Illustration of RSAM.

Specifically, RSAM dynamically upsamples and downsamples the input according to the scale factor. Following this, two sub-branches are created to accommodate each additional scale of the input. The rescaled feature maps in all branches are then scanned by a 3 × 3 convolution to acquire multi-scale features. Subsequently, RSAM generates a total of three sets of LR-HR mapping information. Assuming the scale factor is 2×, the generated mapping pairs are 2×, 2×, and 4×, as depicted in Figure 4. Finally, attention maps for three scales are generated along spatial dimensions, and features from each branch are adjusted and enhanced for the LR-HR mapping mode. Further details are provided in Figure 5. And the entire process is formulated as follows:

(3) $$F_{sf}^{up}=\mu \left(F\right)$$

(4) $$F_{sf}^{dn}=\eta \left(F\right)$$

(5) $$M_{sf}^{main}=\sigma \left(c\left[AdpMixedPool\left(cr\left(F,R\right)\right)\right]\right)$$

(6) $$M_{sf}^{up}=\sigma (c\left[AdpMixedPool_{\eta }\left(cr\left(F_{sf}^{up},R\right)\right]\right)$$

(7) $$M_{sf}^{dn}=\sigma (c\left[AdpMixedPool_{\mu }\left(cr\left(F_{sf}^{dn},R\right)\right)\right]$$

(8) $$F_{sf}^{RSA}=c\left(\left[M_{sf}^{main}⊗F⊕M_{sf}^{up}⊗\eta \left(F_{sf}^{up}\right)⊕M_{sf}^{dn}⊗\mu \left(F_{sf}^{dn}\right)\right],R\right)$$

The input feature map is $F\in R^{C\times H\times W}$. Then, RSAM resizes the input according to the scale factor. And the upsampled and downsampled *F*s are represented by $F_{sf}^{up}\in R^{C\times (H\times sf)\times (W\times sf)}$ and $F_{sf}^{dn}\in R^{C\times (H/sf)\times (W/sf)}$, respectively, obtained through upsampling μ and downsampling η via bilinear interpolation, where sf denotes the target scale factor of the sampling operation (e.g., 2×, 4×, or 8× in our experiments). After a 3×3 convolution layer and ReLU activation cr(·), each pathway employs adaptive average- and max-pooling operations with scale adjustment, followed by concatenation along the channel axis with scale recovering ($AdpMixedPool_{\mu }\left(\cdot \right)$, $AdpMixedPool_{\mu }\left(\cdot \right)$ and $AdpMixedPool_{\eta }\left(\cdot \right)$). Subsequently, a 1×1 convolution layer c(·) and a Sigmoid operation σ generate 2D spatial attention maps $M_{sf}^{main}\in R^{H\times W}$, $M_{sf}^{up}\in R^{H\times W}$, and $M_{sf}^{dn}\in R^{H\times W}$ for each pathway. Element-wise multiplication ⊗ is applied, and the output of each branch is fused via element-wise addition ⊕, resulting in the refined output $F_{sf}^{RSA}$ of the input *F*, after recovering the dimension by a 1×1 convolution c(·). The adjustment in the bandwidth of the channel is finished by the first and last convolution layers. *R* denotes the ratio of the elastic adjuster. The output channel of the first convolution operation is C×R, and the last convolution layer restores the channel to that of the input.

### 3.5. Rich Channel Attention Module (RCAM)

**Figure 6:**
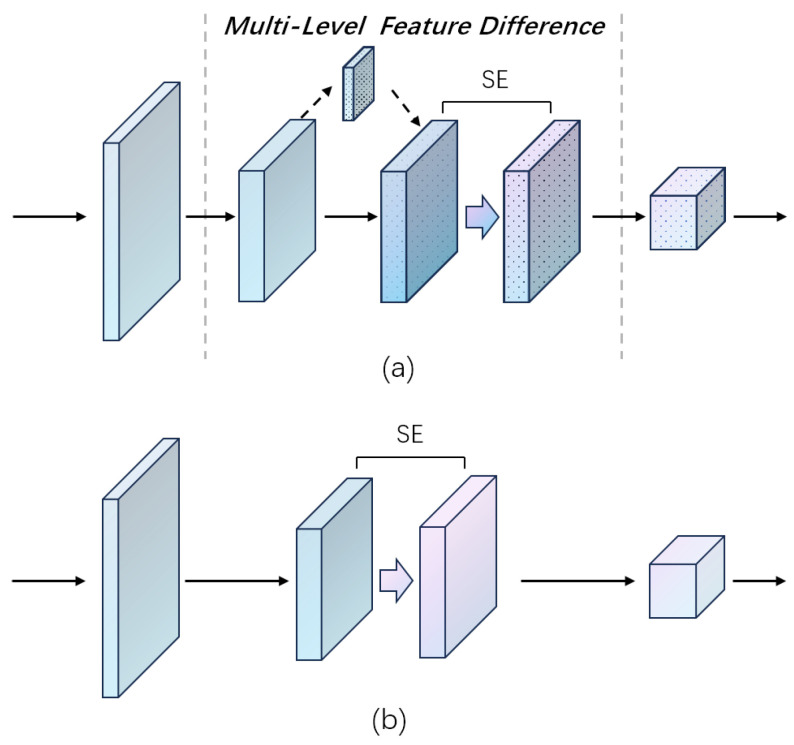
Comparison of RCAM and other channel attention modules. (**a**) RCAM (ours); (**b**) other channel attention modules.

**Figure 7:**
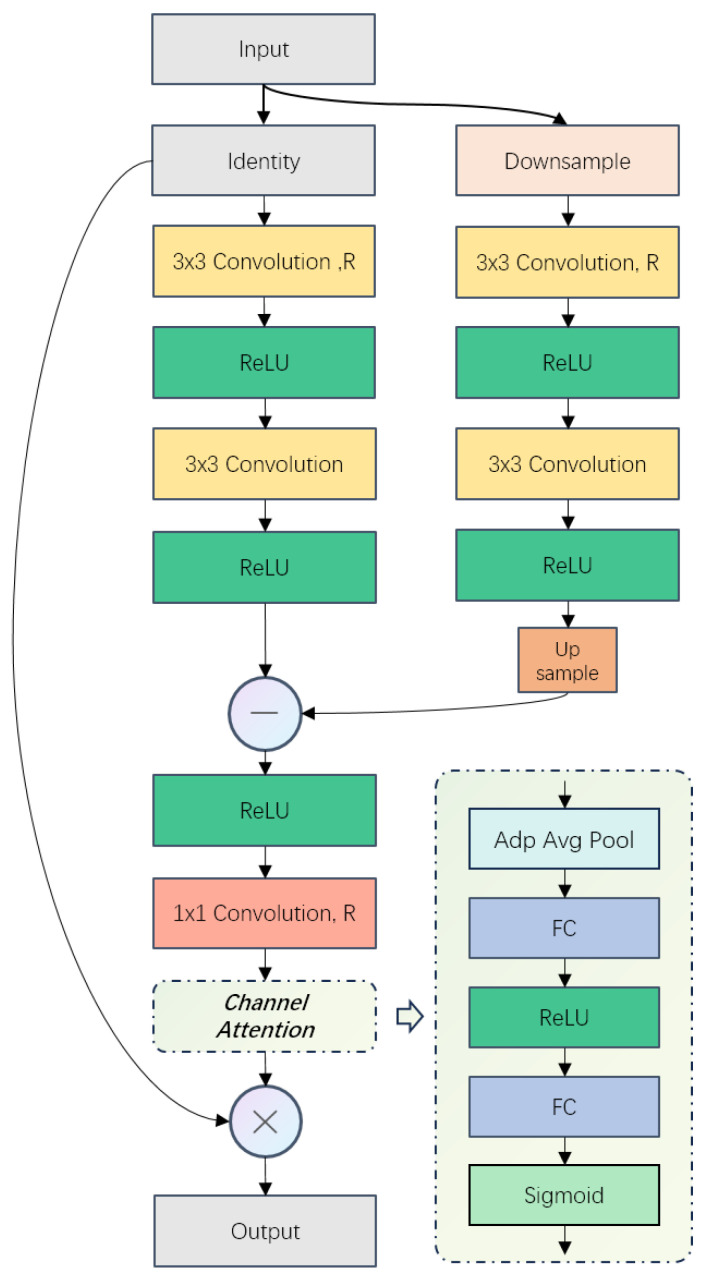
Illustration of RCAM.

After completing the learning process of upscaling, during the denoising stage, RCAM focuses on pixels causing differences to their ground-truth images after rescaling to the same size, aiming to effectively capture such features to minimize $D_{HR}$ and highlight these features along the channel dimensions during channel changes. Similar to RSAM, RCAM creates a sub-branch for downscaling the input. Additionally, in this sub-branch, the number of channels is also adjusted alongside the scale, as differences may arise from both rescale and convolution operations. This sub-branch establishes a middle level between layers for learning multi-level features interactively. Moreover, from a super-resolution perspective, this sub-branch offers an intermediate layer for progressive sampling, enhancing reconstruction quality under high-scale factors, a capability not achieved by other channel-related attention modules (e.g., CAM and SE) (Figure 6). Further details are provided in Figure 7.

And the entire RCAM process is formulated as follows:

(9) $$F^{Main}=F$$

(10) $$F^{Sub}=\eta F$$

(11) $$f_{cr3}^{Main}=cr\left(cr\left(cr\left(F^{Main},R\right)\right)\right)$$

(12) $$f_{cr3}^{Sub}=cr\left(cr\left(cr\left(F^{sub}\right)\right)\right)$$

(13) $$f^{D}=c\left(\left[f_{cr3}^{Main}⊖\mu f_{cr3}^{Sub}\right],R\right)$$

(14) $$M^{RCA}=\sigma (FC(ReLU\left(FC\left(AvgPool_{1\times 1}\left(f^{D}\right)\right)\right)$$

(15) $$F^{RCA}=F⊗M^{RCA}$$

In our experiment, RCAM resizes the input feature $F\in R^{C\times H\times W}$ and utilizes a Convolution ReLU (CR) layer to create $F^{Sub}\in R^{C/2\times H/2\times W/2}$ for the sub-branch. Subsequently, a CR layer (cr(·)) is employed for feature extraction, adjusting the scale and channel number to match the feature maps of the main pathway. Simultaneously, $F^{Main}$ undergoes filtering by three CR layers to retain features at the original resolution. The intermediate feature maps of each pathway are denoted as $f_{cr3}^{Main}$ and $f_{cr3}^{Sub}$ respectively. Following this, features that exhibit significant differences (or noise) $f^{D}$ when resolution changes are obtained via an element-wise subtraction operation ⊖. Subsequently, spatial dimensions are compressed to 1×1 using adaptive average pooling $AvgPool_{1\times 1}\left(\cdot \right)$, followed by FC-ReLU-FC layers and the Sigmoid function σ to generate attention maps $M^{RCA}$ of $f^{D}$, resulting in $F^{FCA}$ as the adjusted output of the input *F*. Like RSAM, the adjustment in the bandwidth of the channel is finished by the first and last convolution layers. *R* denotes the ratio of the elastic adjuster. The output channel of the first convolution operation is C×R, and the last convolution layer restores the channel to that of the input.

### 3.6. REMA-Based Backbone

**Figure 8:**
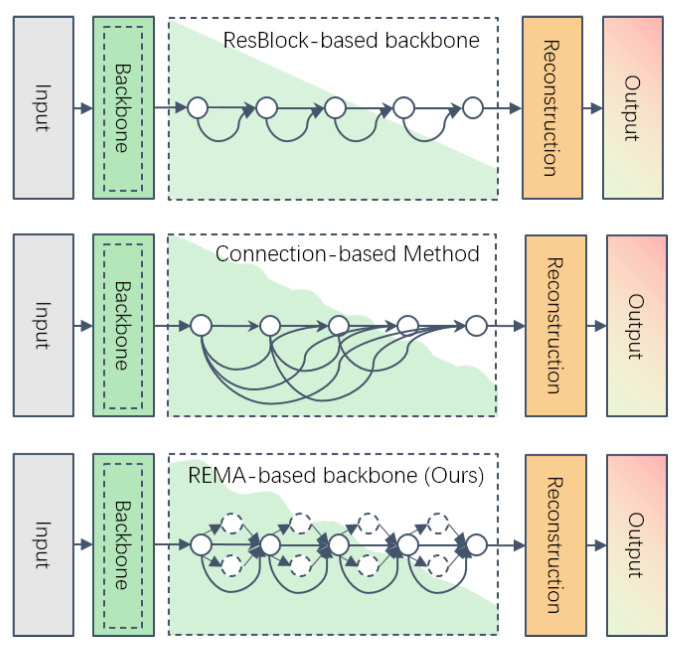
Comparison of REMA-based Backbone and Other Methods. The trend of scale changes in the backbone is denoted by green waves.

As discussed, efficiently extracting features at various scales in the backbone is crucial for CNN-based SISR algorithms. In REMA-SRNet, REMA is integrated into the residual block (REMA ResBlock) in the backbone. As depicted in Figure 8, during the feature extraction process, input features in each residual block layer pass through and iteratively generate LR-HR image pairs within the layer. Compared with connection-based algorithms that achieve features from various scales through connection transfer, the REMA-based backbone provides richer features at diverse resolutions.

## 4. Experiments

### 4.1. Implementation Details and Datasets

To assess the effectiveness of Rich Structure, REMA, and REMA-SRNet, we employ images from [46] for training and validation, following DIV2K’s default split. Evaluation metrics include the peak signal-to-noise ratio (PSNR, dB) and structural similarity (SSIM), computed in the RGB space, where higher values indicate superior reconstruction. The best models are selected based on the highest PSNR + SSIM on the validation set of DIV2K and evaluated on five commonly used datasets (BSDS100 [47], Set14 [48], Set5 [49], Manga109 [50], and Urban100 [51]), and an additional three datasets (Historical [52], PIRM [53], and General100 [26]) for comprehensive study, under upscaling factors of 2×, 4×, and 8×, respectively. HR images are center-cropped to 256×256 patches, downscaled via bicubic interpolation to generate LR image pairs for training and testing, without any data augmentation. Optimization employs Adam with an initial learning rate of 0.0001, halved every 50 epochs, $\beta_{1}$ set to 0.9, $\beta_{2}$ set to 0.999, and ϵ set to $10^{-6}$. The batch size is set to 1, and training lasts 300 epochs, using PyTorch 2.0.0 on a desktop with an Intel I5-8600 CPU, 64GB RAM, and NVIDIA GTX 3090 GPU. The training loss function is L1 loss.

(16) $$L^{\ell_{1}}\left(P\right)=\frac{1}{N}\sum_{p\in P}\left|x\left(p\right)-y\left(p\right)\right|$$

where *P* represents the calculated area, and *p* denotes the pixel’s position within area *P*. The pixel values at position *p* in both the prediction area x(p) and the ground truth map GT area y(p) are taken into account.

### 4.2. Evaluation Metrics

We evaluate SR images using two widely used metrics: the peak signal-to-noise ratio (PSNR) and structural similarity (SSIM). PSNR serves as an objective metric to assess image quality and measure the degree of difference between an original image and a compressed or distorted version. The PSNR calculation relies on mean square error (MSE), quantifying the squared differences between corresponding pixels in the original and reconstructed images. The formula for PSNR is as follows:

(17) $$M_{MSE}=\frac{1}{WH}\sum_{i=0}^{W-1}\sum_{j=0}^{H-1}{\left[X\left(i,j\right)-Y\left(i,j\right)\right]}^{2}$$

(18) $$PSNR=10\;\log_{10}\left(\frac{X_{MAX}^{2}}{M_{MSE}}\right)$$

where *W* and *H* are the width and height of the image, (i,j) represent pixel positions, and *X* and *Y* denote the super-resolved image and the ground-truth image, respectively. $X_{MAX}$ is the maximum pixel value range, and $M_{MSE}$ stands for mean square error. Higher PSNR values indicate lower distortion and better image quality, typically ranging from 20 to 50. PSNR values exceeding 30 dB are generally considered indicative of good image quality. Recognizing that PSNR is a limited indicator that fails to capture human subjective perception of images, we also utilize SSIM as an evaluation index. SSIM accounts for contrast, brightness, and structural similarity. The calculation for the SSIM value at the pixel position, *p*, is as follows:

(19) $$SSIM\left(p\right)=\frac{2\mu_{x}\mu_{y}+C_{1}}{\mu_{x}^{2}+\mu_{y}^{2}+C_{1}}\cdot \frac{2\sigma_{xy}+C_{2}}{\sigma_{x}^{2}+\sigma_{y}^{2}+C_{2}}$$

Here, $\mu_{x}$, $\mu_{y}$,$\sigma_{x}$, $\sigma_{y}$, and $\sigma_{xy}$ denote the mean, standard deviation, and covariance of pixels at position p in the prediction map and the true value map. Constants $C_{1}$ and $C_{2}$ are included to prevent division by zero. The SSIM value falls within the range of (0,1), with values closer to 1 indicating a superior HR reconstruction effect.

### 4.3. Ablation Studies

In this section, ablation studies are conducted to verify the effectiveness of each part of REMA. The experiments span networks with various settings, scale factors, and integration positions, as well as comparisons with other attention modules in REMA-SRNet and other SISR networks. Meanwhile, the effectiveness of Rich Structure is verified by comparing it with REMA using Inception- and ResNeXt-like structures. Furthermore, the impact of parameter count on performance and robustness is discussed based on the experimental results.

The baseline model in our experiment is the proposed modified EDSR (replacing REMA ResBlock with residual blocks in REMA-SRNet). Specifically, the pixel shuffle layer is replaced by bilinear upsampling followed by 3 × 3 convolutions and Leaky ReLU layers, connected with the bilinear-upscaled LR input. To validate the proposed methods, we employ two sets of network configurations: default (64-16-64) as REMA-SRNet and alternative (40-16-40) as REMA-SRNet-M. The adjuster ratio R is set to 1 by default. For 2×, 4×, and 8× reconstruction, the number of reconstruction layers is 1, 2, and 3, respectively.

In all experiments, robustness is evaluated across all eight datasets. The Historical dataset specifically assesses the module’s capability in handling out-of-distribution (OOD) samples. Additionally, two sets of network configurations mentioned above (#C_In is 40 or 64) are employed to test module compatibility with varying complexities of input features.

#### 4.3.1. Study of REMA in the Backbone

Figure 3 illustrates the utilization of REMA within a residual block of the backbone networks. To analyze the effectiveness of REMA, except the baseline, five models were constructed: RSAM, RCAM, RSAM-RCAM, RCAM-RSAM, and REMA, and they represent the model with RSAM and RCAM, and employ them together in parallel, respectively. The results of all the above ablation experiments are shown in Table 2.

The results indicate that employing only RSAM in the backbone enhances PSNR and SSIM across most datasets, except for the Set14 and Historical datasets when the input tensor has 40 channels. However, RCAM also underperforms in the Historical dataset, attributed to significant differences between the Historical dataset images and the distribution of training datasets. Configuring them in parallel (REMA) boosts performance across most datasets. Moreover, with a 64-channel input tensor, all models show significant performance improvements. Notably, using RSAM and RCAM separately substantially mitigates the performance reduction issue in the Historical dataset. Consequently, the backbone of REMA demonstrates performance improvements in the Historical dataset. Overall, these results affirm the effectiveness of our method.

#### 4.3.2. Study of Rich Structure

The study of Rich Structure, along with REMA throughout subsequent experiments, is examined. To verify the effectiveness of Rich Structure and REMA, we initially compared REMA with other attention modules. This allows us to identify the key factors influencing a plug-and-play module and demonstrate the superiority of Rich Structure and REMA. Additionally, we designed ResNeXt and Inception versions of REMA to highlight the advantages of Rich Structure in terms of compatibility and flexibility compared to other popular module structures.

#### 4.3.3. Comparison with Other Attention Modules

We compared the performance of REMA with other attention modules, including CBAM, SE, CA, and BAM, which were employed in the same way as REMA. Our experiment includes results for 40- and 64-channel input. To ensure a fair comparison, we set the dimension reduction to 1 (C/r, r = 1), meaning no channel compression is applied. The results are presented in Table 3.

The results indicate that for the 40-channel input, there is no significant difference in the performance of REMA with other attention modules, except CBAM. However, for the 64-channel input, REMA outperforms other attention modules. Furthermore, comparing the overall improvement when changing the number of channels from 40 to 64, BAM and REMA show much higher performance than other attention modules in the experiment, as discussed in the next section. For the Historical dataset, except for REMA, there is a reduction in performance after integrating other attention modules into the residual block.

#### 4.3.4. Study of Plain, Multi-Branch, and Rich Structure

To elucidate the performance increment difference with increasing input complexity, we analyze these attention modules from a global structural perspective. According to Table 3, modules with a multi-branch structure exhibit a greater performance increase with the rise in input complexity compared to plain structures, except for CA. The primary distinction among these modules lies in their cardinality: 1 for plain modules (SE and CBAM), and 2, 2, and 5 for CA, BAM, and REMA, respectively. Based on the results, cardinality is positively correlated with the overall performance of modules for a 64-channel input. Thus, cardinality is an influential factor relating to module compatibility, and higher cardinality will enhance the module’s performance with the growth in input complexity.

However, cardinality is not the sole factor influencing performance. When comparing the results of CA and BAM, both with a cardinality of two, there exists a performance gap for the 64-channel input. The main difference lies in the in-branch bandwidth. In fact, CA also employs a split–transform–aggregate structure similar to Inception-like blocks. The distinction is that CA splits the features (C×H×W) along H and W rather than C, as shown in Figure 1b, while BAM and REMA directly map the complete input to branches. This implies that the in-branch features are less informative in CA compared to BAM and REMA.

Comparing BAM and REMA, both modules generate spatial and channel attention. The difference lies in our proposed algorithm, which not only enhances SR-related feature representation but also generates richer multi-scale and multi-level features compared to BAM. This is because BAM is a size-oriented module, balancing performance and module size, resulting in limited room and more constraints for algorithm design. Our proposed Rich Structure is designed to overcome this limitation. We will delve into this topic in the following section. Therefore, in-branch feature richness and task-related algorithms are other influential factors. The richness is defined by the channel bandwidth of the in-branch features and the diversity of features(multi-scale and multi-level features).

#### 4.3.5. Study of the Elastic Adjuster

For further investigation, we conducted an experiment to analyze the influence of overall channel bandwidth on performance. The overall channel bandwidth of modules with plain structures, multi-branch structures, and our proposed Rich Structure differs significantly, with the plain structure being much slimmer than the others. We redesigned these modules, replacing dimension reduction with the elastic adjuster (C×R), where R is set to 3, indicating a widened channel bandwidth by 3 times to determine how bandwidth affects the performance and to verify the effectiveness of the elastic adjuster in different attention modules. The results are presented in Table 4, and there is a dedicated section for this independent experiment in REMA in the following.

The results show that for the 40-channel input, the redesigned wider CBAM and SE exhibit improvements on most datasets, bringing their performances close to those of the original CA and BAM, which performed better than them previously. This underscores the significance of the in-branch feature bandwidth of the channel as a key performance-related factor, which ultimately affects the overall module’s width. These results highlight how plain structures and dimension-reduction components, realized by bottleneck structures, actually limit their potential in performance, proving the effectiveness of the proposed elastic adjuster in enhancing performance when needed alongside the Rich Structure under certain conditions. However, for the 64-channel input, a reduction occurs in wider modules, except for BAM. For BAM, the redesign results in improvements for half of the datasets and reductions on others, with overall performance close to the original for the 64-channel input. This indicates a limit to increasing in-branch channel bandwidth for further performance gains.

#### 4.3.6. Study of the Elastic Adjuster in REMA

To analyze the effect of in-branch channel bandwidth in REMA, experiments are conducted. Specifically, in the experiments, the elastic adjuster’s ratio was varied from 0.5 to 1.5, and the performances of R∈[0.5,1) and R∈[1,1.5], representing the size-oriented and performance-oriented modes of REMA, were compared. The results are shown in Table 5.

The results indicate that the overall performance of size-oriented REMA is lower than the performance-oriented one for the 40-channel input, showing the same trend as the widened versions of other attention modules. However, for the 64-channel input, different from other widened attention modules, A can still benefit from the increased bandwidth for some datasets, including BSDS100, Mange109, Set14, and Urban100. Additionally, the performance gap between the lowest and highest values for the 64-channel input is not large, proving that REMA can ensure flexibility to meet different task requirements by switching the elastic adjuster.

There is still a limit to achieving more performance through parameter exchange. This limitation may stem from two aspects: input complexity and task-specific algorithms. Regarding the former, comparing the results of 40_1.5 and 64_0.6, it can be observed that they have similar numbers of parameters, yet 64_0.6 performs significantly better than 40_1.5, with the only difference being the number of input channels. This illustrates one of the reasons why models with more parameters do not always yield higher performance and why a plug-and-play module works in one network but not in another.

Concerning the latter, comparing REMA with the widened version of BAM (64_1.2), both having a multi-branch structure with the elastic adjuster and similar overall channel bandwidth (BAM: 2×3, REMA: 5×1.2), REMA outperforms BAM on all datasets. Furthermore, the results of R∈[0.5,1) and R∈[1,1.5] demonstrate that a more effective parameter exchange provides extra robustness on different datasets, although models with fewer parameters may perform better on certain datasets.

#### 4.3.7. Size-Oriented vs. Performance-Oriented

In order to investigate how lightweight structures affect performance further, we compare Rich Structure (copy/rescale–transform–aggregate) with other size-oriented multi-branch designs. Specifically, we redesign REMA in Inception (split–transform–concatenate) and ResNeXt (split–transform–aggregate) styles. The split operation is achieved by setting the elastic adjuster to be 1/3 in RSAM and 1/2 in RCAM to maintain the overall bandwidth the same as the input feature. Additionally, the main difference between the Inception and ResNeXt versions lies in the topology of each transforming branch, whereas in ResNeXt, they are the same. Hence, we propose an extra version of it to maintain multi-scale and multi-level feature fusion as used in REMA, to verify their effectiveness.

To comprehensively discuss the parameter efficiency of size-oriented and performance-oriented structures, we also consider the scale factor for two reasons. Firstly, from the SR task perspective, a higher scale factor makes SR inference more challenging. From the network perspective, as the scale factor increases, the network becomes more prone to overfitting since we generate training data by downsampling the ground-truth image at the target scale factor rate. Consequently, the input patch becomes very small at 8× (32×32), potentially leading to overfitting for a module that performs well at 2× and 4×. In other words, 2×, 4×, and 8× represent three situations, ranging from low to high difficulty for every parameter that influences performance. Additionally, performance on the Historical dataset receives more attention as it represents an out-of-distribution (OOD) scenario. Therefore, we use these factors to test the module’s compatibility and robustness, with the experiment results presented in Table 6.

According to the results, Rich Structure outperforms other versions of REMA. Although the performances of Inception and ResNext_MS may be close to the Rich Structure version of REMA in certain datasets or certain upscale ratios, overall, the Rich Structure version demonstrates the best capability across different datasets and networks, with less likelihood of overfitting. Moreover, comparing ResNeXt_MS shows better performance than ResNeXt under 2× and 4×, and their results are comparable under 8x, highlighting the effectiveness of the multi-scale and multi-level feature fusion strategy in REMA. These findings demonstrate the higher compatibility and robustness of our method compared to other popular size-oriented multi-branch structures when applied in the backbone. Again, the results demonstrate that extra effective parameters can exchange and provide more robustness under different scale factors.

#### 4.3.8. Study of REMA in the Reconstruction Layer

Additionally, given the application of REMA in reconstruction layers at high-scale factors, experiments are conducted at scale factors of 4× and 8×. Figure 3 illustrates the implementation of REMA in the reconstruction layer, with corresponding results shown in Table 7.

In summary, the significance of REMA in reconstruction blocks increases with larger-scale factors. At 4×, it results in a performance decline in most datasets, leading to its exclusion from REMA-SRNet under 2× and 4×. However, at 8×, there is an improvement in most datasets when used in reconstruction for the 64-channel input. However, for the 64-channel input, the overall enhancement is less evident. Hence, REMA in reconstruction layers improves performance at high-scale ratios under specific conditions.

#### 4.3.9. Study of REMA in Other SISR Network

For further investigation, we incorporate REMA into UNet-SR, a super-resolution network based on the image segmentation network U-Net. UNet-SR employs skip connections for encoder–reconstruction feature fusion, enhancing reconstitution quality. We utilize this setup to assess REMA’s effectiveness in other networks and evaluate its impact on performance when integrated into skip connections. This extends the experiments beyond the backbone and reconstruction layers, as skip connections were not used in REMA-SRNet for varying depth feature fusion. Results are summarized in Table 8.

The results show that the performance of REMA, when added to the skip connection, surpasses other attention modules at the same position, indicating that REMA remains effective in various SR models and positions. In fact, the number of input channels gradually expands, layer by layer, as it progresses from shallow to deep within the skip connections of UNet-SR. Thus, this also suggests that Rich Structure’s advantage becomes more pronounced when handling inputs with more filters, outperforming other attention modules.

#### 4.3.10. Comparison with Other Comparative Methods

To comprehensively evaluate our methods, we compare REMA-SRNet (R = 1) with other SISR methods, employing similar approaches such as residual, recursive, and multi-branch learning, as well as attention-based SR networks. Our experiments encompass both lightweight and large models, including VDSR [5], ESPCN [25], RCAN [27], PAN [18], A2N [36], DRLN [10], RCAN [27], ESRGCNN [29], SwinIR [16], NLSN [20], and UNet-SR [30].

Table 9 displays the quantitative results for various scaling factors. In summary, compared to other SOTAs, REMA outperforms other methods for 2×, 4×, and 8× upscaling on benchmark datasets, showcasing the effectiveness of REMA-SRNet. Further research should address the parameter-efficiency perspective when discussing trends in results.

**Figure 9:**
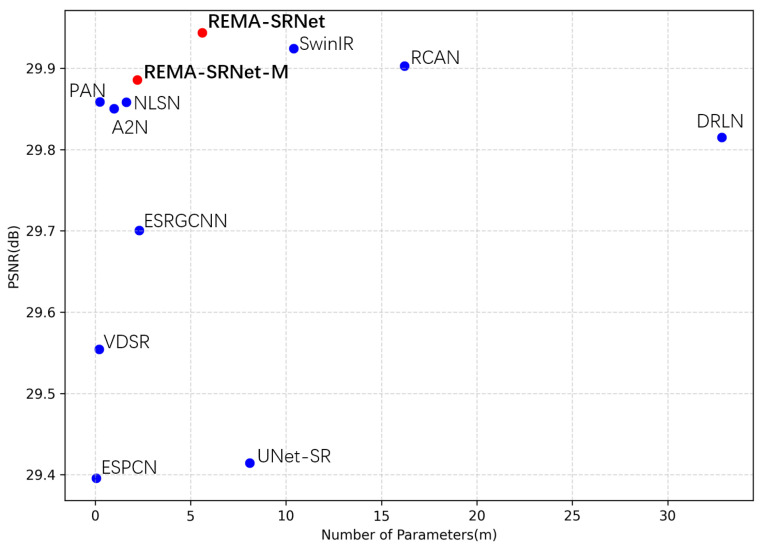
Performance comparison between REMA-SRNet and other SISR methods on BSDS100 (2×). Our algorithms are highlighted in red.

The results indicate that methods with large sizes do not necessarily equate to high performance. In fact, size and performance show some positive correlation at 4×. As explained earlier, this is due to complex models being prone to overfitting as the complexity of the training data decreases with the increasing scale factor. For instance, RCAN and DRLN may achieve better results on certain datasets at 2× and 4× but perform worse than lightweight models like PAN and A2N at 8× due to overfitting. Conversely, while lightweight models may excel in specific scale factors or datasets, they may be insufficient for performance-prioritized tasks or broad compatibility requirements. Thus, parameter efficiency not only achieves intermediate results with few parameters but also attains optimal results while maintaining the overall model size. Among the models tested, only REMA-SRNet and SwinIR achieve this balance. REMA-SRNet generally outperforms SwinIR while using only 60% of its parameters (Figure 9).

### 4.4. Visual Comparison of Different Models

**Figure 10:**
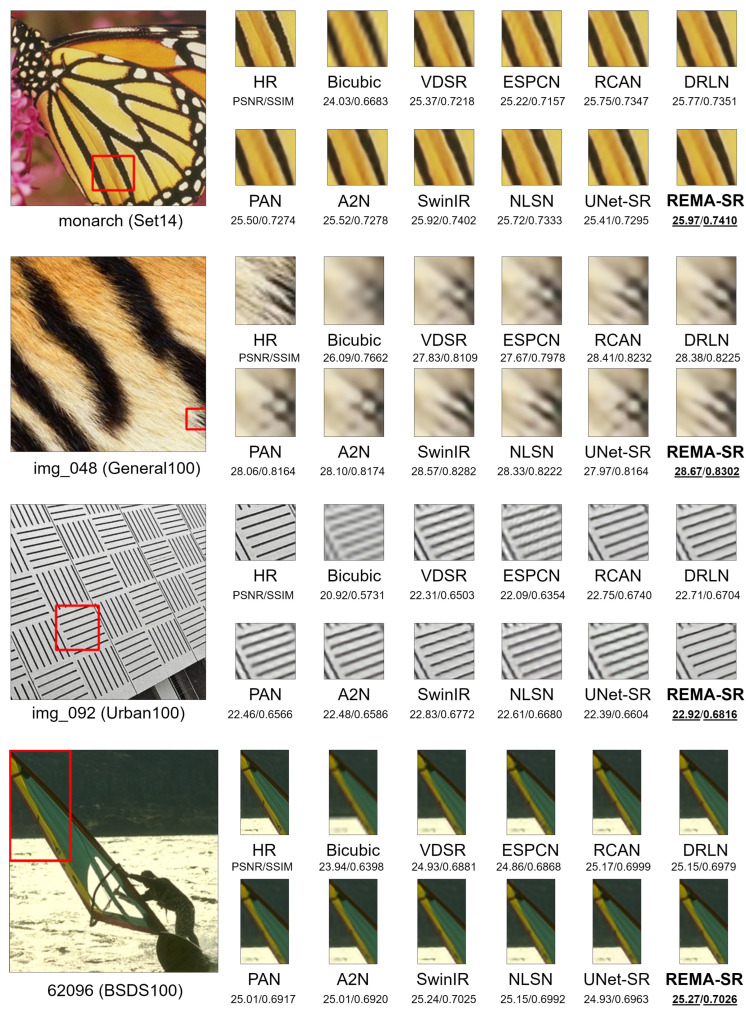
Subjective quality assessment for 4× upscaling on the general images from four datasets. The best results are bold and underlined.

We selected reconstructed images from the Urban100, BSDS100, General100, and SET14 datasets to compare reconstruction details. Figure 10 illustrates the HR effects of REMA-SRNet and other methods, highlighting smoother lines, the preservation of fine details, and improved textures in the reconstructed images. Specifically, the textures in the super-resolved images ’img_048’ and ’img_092’ by REMA-SRNet are more accurate, and the lines in ’monarch’ and ’62096’ are sharper compared to other methods.

## 5. Conclusions

To address the challenge of detail preservation in SISR tasks, we propose a plug-and-play attention module called REMA. The core component, Rich Structure, is proposed based on extensive research into how different module structures impact size, compatibility, and performance. This allows REMA to seamlessly transition between being size-oriented and performance-oriented, depending on the specific requirements of the task. We separate the SR process into two steps: upsampling and denoising, with RSAM and RCAM designed to focus on the key factors in each step, respectively. Building on Rich Structure, we propose RSAM and RCAM. RSAM focuses on the mutual dependency of multiple LR and HR pairs, as well as multi-scale features, while RCAM uses interactive learning to emphasize key features, enhancing detail and noise differentiation and generating intermediate features for multi-level feature fusion. Thus, with RSAM and RCAM, REMA enhances the SISR process and the performance of deep learning-based networks by simultaneously improving long-range dependency learning. Together, these components alleviate issues of algorithm flexibility and detail preservation.

Extensive experiments validate the effectiveness of REMA, showing significant improvements in performance and compatibility compared to other attention modules. Additionally, REMA-SRNet demonstrates superiority over other SISR networks. Our investigations into module compatibility reveal a correlation between cardinality, in-branch feature bandwidth, and compatibility. Further analysis indicates that networks with high effective parameter counts exhibit enhanced robustness across various datasets and scale factors.

Future work will continue to explore factors influencing the performance and robustness of modules and aim to improve super-resolution accuracy. We plan to introduce more metrics and explore higher super-resolution ratios, such as 16×. Our goal is to develop a plug-and-play module that can automatically adjust its structure and complexity, ensuring cost efficiency and reducing the need for manual parameter tuning to meet diverse requirements.

## Figures and Tables

**Table 1:** Implications of nouns, abbreviations, and symbols.

Abbreviation/Symbols	Implication
REMA	rich elastic mixed attention
REMA-SRNet	REMA-based SR network
RSAM	rich spatial attention module
RCAM	rich channel attention module
AvgPool	average pooling
Adp Avg pool	adaptive average pooling
Adp mixed pool	adaptive average⊕maximum pooling
SF	scale factor
R	The ratio of the elastic adjuster
Conv	convolution
FC	fully connected layer
Concate	Concatenate
⊗	element-wise multiplication
⊕	element-wise addition
⊖	element-wise subtraction

**Table 2:** The effect of each part of REMA in the backbone (4×). #C_In denotes the input tensor’s channel count. Numerical comparisons maintain precision to 12 decimal places, with the top two results highlighted in red and blue.

MODEL	#C_In	BSDS100		General100		Historical		Manga109		PIRM		SET14		SET5		Urban100	
		PSNR	SSIM	PSNR	SSIM	PSNR	SSIM	PSNR	SSIM	PSNR	SSIM	PSNR	SSIM	PSNR	SSIM	PSNR	SSIM
Baseline	40	25.17	0.6993	28.45	0.8257	22.04	0.6696	26.36	0.8413	28.16	0.8918	25.84	0.7374	29.78	0.8651	22.73	0.6711
RCAM	40	25.20	0.7002	28.52	0.8275	22.01	0.6678	26.48	0.8437	28.21	0.8930	25.85	0.7387	29.90	0.8667	22.84	0.6756
RSAM	40	25.20	0.7009	28.50	0.8272	22.00	0.6703	26.44	0.8434	28.32	0.8941	25.84	0.7385	29.90	0.8674	22.74	0.6736
**REMA**	40	25.23	0.7019	28.54	0.8285	22.00	0.6707	26.53	0.8457	28.22	0.8930	25.88	0.7394	30.01	0.8689	22.86	0.6775
Baseline	64	25.22	0.7005	28.52	0.8263	22.03	0.6701	26.44	0.8435	28.29	0.8929	25.83	0.7376	29.88	0.8676	22.82	0.6753
RCAM	64	25.24	0.7022	28.63	0.8297	22.03	0.6721	26.61	0.8475	28.35	0.8963	25.89	0.7401	30.04	0.8701	22.88	0.6803
RSAM	64	25.24	0.7023	28.63	0.8294	22.03	0.6718	26.61	0.8473	28.43	0.8971	25.89	0.7400	29.99	0.8697	22.90	0.6801
**REMA**	64	25.27	0.7026	28.67	0.8302	22.05	0.6722	26.65	0.8484	28.41	0.8962	25.97	0.7410	30.04	0.8700	22.92	0.6816

**Table 3:** Performance comparison in ResBlock between REMA and other attention modules. #C_In denotes the input tensor’s channel count. Numerical comparisons maintain precision to 12 decimal places, with the top two results highlighted in red and blue.

MODEL	#C_in	BSDS100		General100		Historical		Manga109		PIRM		SET14		SET5		Urban100	
		PSNR	SSIM	PSNR	SSIM	PSNR	SSIM	PSNR	SSIM	PSNR	SSIM	PSNR	SSIM	PSNR	SSIM	PSNR	SSIM
CBAM [23]	40	25.20	0.6999	28.49	0.8270	22.01	0.6687	26.44	0.8435	28.17	0.8935	25.83	0.7370	29.89	0.8663	22.81	0.6755
SE [31]	40	25.20	0.7010	28.51	0.8280	21.98	0.6695	26.51	0.8459	28.24	0.8931	25.87	0.7397	29.97	0.8686	22.78	0.6757
CA [32]	40	25.24	0.7022	28.51	0.8276	21.96	0.6675	26.54	0.8457	28.12	0.8905	25.85	0.7394	29.96	0.8684	22.79	0.6768
BAM [19]	40	25.22	0.7016	28.57	0.8284	21.96	0.6692	26.54	0.8464	28.30	0.8936	25.88	0.7396	29.95	0.8690	22.85	0.6775
**REMA**	40	25.23	0.7019	28.54	0.8285	22.00	0.6707	26.53	0.8457	28.22	0.8930	25.88	0.7394	30.01	0.8689	22.86	0.6775
CBAM [23]	64	25.21	0.7004	28.57	0.8281	22.01	0.6694	26.48	0.8443	28.30	0.8957	25.86	0.7384	29.87	0.8676	22.81	0.6767
SE [31]	64	25.24	0.7016	28.61	0.8292	22.02	0.6714	26.55	0.8464	28.41	0.8961	25.92	0.7403	29.96	0.8691	22.89	0.6796
CA [32]	64	25.22	0.7014	28.56	0.8281	22.01	0.6700	26.53	0.8457	28.45	0.8959	25.88	0.7397	29.97	0.8689	22.80	0.6780
BAM [19]	64	25.24	0.7016	28.64	0.8294	22.01	0.6698	26.61	0.8476	28.36	0.8957	25.90	0.7400	30.01	0.8691	22.90	0.6804
**REMA**	64	25.27	0.7026	28.67	0.8302	22.05	0.6722	26.65	0.8484	28.41	0.8962	25.97	0.7410	30.04	0.8700	22.92	0.6816

**Table 4:** Performance comparison between widened attention modules #C_In denotes the input tensor’s channel count. _wide denotes the modules with widened channel bandwidth (×3) by the elastic adjuster, and R denotes the ratio of the elastic adjuster. The numerical comparisons are accurate to 12 decimal places, with the best result highlighted in red.

MODEL	#C_In	R	BSDS100		General100		Historical		Manga109		PIRM		SET14		SET5		Urban100	
			PSNR	SSIM	PSNR	SSIM	PSNR	SSIM	PSNR	SSIM	PSNR	SSIM	PSNR	SSIM	PSNR	SSIM	PSNR	SSIM
CBAM [23]	40	1	25.20	0.6999	28.49	0.8270	22.01	0.6687	26.44	0.8435	28.17	0.8935	25.83	0.7370	29.89	0.8663	22.81	0.6755
CBAM_wide	40	3	25.22	0.7014	28.52	0.8279	22.01	0.6707	26.47	0.8439	28.12	0.8931	25.87	0.7392	29.98	0.8686	22.81	0.6748
SE [31]	40	1	25.20	0.7010	28.51	0.8280	21.98	0.6695	26.51	0.8459	28.24	0.8931	25.87	0.7397	29.97	0.8686	22.78	0.6757
SE_wide	40	3	25.21	0.7013	28.54	0.8279	22.02	0.6711	26.49	0.8451	28.30	0.8935	25.89	0.7395	29.87	0.8673	22.80	0.6757
CA [32]	40	1	25.24	0.7022	28.51	0.8276	21.96	0.6675	26.54	0.8457	28.12	0.8905	25.85	0.7394	29.96	0.8684	22.79	0.6768
CA_wide	40	3	25.22	0.7014	28.58	0.8285	22.00	0.6677	26.57	0.8457	28.23	0.8933	25.87	0.7394	29.93	0.8678	22.79	0.6744
BAM [19]	40	1	25.22	0.7016	28.57	0.8284	21.96	0.6692	26.54	0.8464	28.30	0.8936	25.88	0.7396	29.95	0.8690	22.85	0.6775
BAM_wide	40	3	25.22	0.7016	28.53	0.8280	22.00	0.6708	26.54	0.8458	28.28	0.8935	25.87	0.7395	29.93	0.8687	22.82	0.6778
CBAM [23]	64	1	25.21	0.7004	28.57	0.8281	22.01	0.6694	26.48	0.8443	28.30	0.8957	25.86	0.7384	29.87	0.8676	22.81	0.6767
CBAM_wide	64	3	25.21	0.6985	28.47	0.8264	21.99	0.6653	26.45	0.8433	28.13	0.8917	25.84	0.7372	29.95	0.8686	22.82	0.6750
SE [31]	64	1	25.24	0.7016	28.61	0.8292	22.02	0.6714	26.55	0.8464	28.41	0.8961	25.92	0.7403	29.96	0.8691	22.89	0.6796
SE_wide	64	3	25.24	0.7005	28.56	0.8277	21.99	0.6682	26.57	0.8457	28.27	0.8926	25.88	0.7387	29.96	0.8684	22.88	0.6785
CA [32]	64	1	25.22	0.7014	28.56	0.8281	22.01	0.6700	26.53	0.8457	28.45	0.8959	25.88	0.7397	29.97	0.8689	22.80	0.6780
CA_wide	64	3	25.19	0.7002	28.51	0.8277	21.91	0.6649	26.49	0.8456	28.11	0.8920	25.83	0.7381	30.00	0.8694	22.77	0.6757
BAM [19]	64	1	25.24	0.7016	28.64	0.8294	22.01	0.6698	26.61	0.8476	28.36	0.8957	25.90	0.7400	30.01	0.8691	22.90	0.6804
BAM_wide	64	3	25.23	0.7016	28.62	0.8292	22.00	0.6703	26.64	0.8483	28.36	0.8953	25.90	0.7400	30.00	0.8693	22.91	0.6813

**Table 5:** The trend of performance changes with different ratios of the elastic adjuster under 4×. #C_in_R denotes the number of channels of the input and the elastic adjuster’s ratio. The results of different input widths are denoted by blue and green. Deeper colors represent higher values.

#C_in_R	#P(M)	BSDS100		General100		Historical		Manga109		PIRM		SET14		SET5		Urban100	
		PSNR	SSIM	PSNR	SSIM	PSNR	SSIM	PSNR	SSIM	PSNR	SSIM	PSNR	SSIM	PSNR	SSIM	PSNR	SSIM
40_0.5	1.24	25.1891	0.7007	28.4632	0.8269	22.0067	0.6701	26.4201	0.8442	28.1106	0.8925	25.8111	0.7380	29.8876	0.8671	22.7261	0.6737
40_0.6	1.42	25.2001	0.7011	28.5101	0.8274	21.9754	0.6696	26.4401	0.8448	28.2500	0.8932	25.8032	0.7382	29.9097	0.8676	22.7979	0.6760
40_0.7	1.60	25.2416	0.7012	28.5283	0.8277	22.0323	0.6700	26.5091	0.8451	28.2527	0.8924	25.9023	0.7389	29.9764	0.8677	22.8515	0.6759
40_0.8	1.80	25.2216	0.7007	28.4686	0.8270	21.9984	0.6697	26.4743	0.8443	28.0599	0.8918	25.8125	0.7378	29.8401	0.8668	22.7955	0.6753
40_0.9	2.01	25.1979	0.7006	28.5478	0.8275	22.0486	0.6709	26.4414	0.8436	28.2761	0.8936	25.8391	0.7382	29.9706	0.8681	22.7844	0.6741
40_1.0	2.23	25.2304	0.7019	28.5352	0.8285	22.0010	0.6707	26.5325	0.8457	28.2203	0.8930	25.8836	0.7394	30.0089	0.8689	22.8582	0.6775
40_1.1	2.46	25.2125	0.7016	28.5024	0.8278	21.9662	0.6700	26.5181	0.8458	28.2002	0.8924	25.8442	0.7393	29.9410	0.8682	22.8043	0.6773
40_1.2	2.71	25.2251	0.7008	28.5075	0.8277	22.0353	0.6707	26.4892	0.8445	28.1908	0.8918	25.8655	0.7385	29.9313	0.8680	22.8066	0.6755
40_1.3	2.96	25.2137	0.7016	28.5226	0.8283	21.9833	0.6703	26.4796	0.8458	28.1377	0.8928	25.8514	0.7390	29.9491	0.8689	22.8251	0.6780
40_1.4	3.23	25.2094	0.7009	28.5167	0.8275	22.0156	0.6689	26.4634	0.8440	28.1621	0.8927	25.8824	0.7389	30.0125	0.8683	22.7892	0.6743
40_1.5	3.51	25.2120	0.7014	28.5226	0.8284	22.0392	0.6720	26.5478	0.8463	28.3206	0.8942	25.9114	0.7402	29.9412	0.8683	22.8124	0.6762
64_0.5	3.17	25.2544	0.7014	28.6561	0.8297	22.0274	0.6702	26.6492	0.8479	28.3847	0.8954	25.9461	0.7404	30.0146	0.8692	22.9101	0.6806
64_0.6	3.58	25.2543	0.7017	28.6454	0.8296	22.0369	0.6712	26.6163	0.8474	28.3465	0.8956	25.9149	0.7400	30.0163	0.8692	22.8879	0.6795
64_0.7	4.02	25.2573	0.7017	28.6285	0.8298	22.0126	0.6706	26.5892	0.8472	28.3369	0.8951	25.9123	0.7400	30.0121	0.8698	22.9066	0.6803
64_0.8	4.55	25.2574	0.7023	28.6499	0.8300	21.9949	0.6703	26.6493	0.8485	28.3641	0.8956	25.9439	0.7408	30.0906	0.8706	22.9113	0.6807
64_0.9	5.05	25.2556	0.7019	28.6552	0.8301	22.0127	0.6709	26.6080	0.8474	28.3579	0.8957	25.9473	0.7405	30.0138	0.8696	22.8951	0.6801
64_1.0	5.68	25.2675	0.7026	28.6692	0.8302	22.0525	0.6722	26.6482	0.8484	28.4064	0.8962	25.9686	0.7410	30.0447	0.8700	22.9186	0.6816
64_1.1	6.23	25.2606	0.7020	28.6570	0.8304	21.9980	0.6713	26.6233	0.8477	28.3300	0.8954	25.9443	0.7407	30.0728	0.8707	22.9230	0.6813
64_1.2	6.82	25.2668	0.7022	28.6752	0.8305	22.0258	0.6708	26.6442	0.8480	28.4011	0.8954	25.9441	0.7409	30.0737	0.8704	22.9175	0.6817
64_1.3	7.52	25.2690	0.7033	28.6565	0.8305	21.9993	0.6720	26.6622	0.8492	28.3611	0.8959	25.9383	0.7414	30.0376	0.8704	22.9244	0.6823
64_1.4	8.16	25.2599	0.7022	28.6557	0.8302	22.0226	0.6715	26.6717	0.8488	28.3695	0.8954	25.9690	0.7409	30.0291	0.8695	22.8993	0.6808
64_1.5	8.96	25.2652	0.7021	28.6402	0.8300	22.0262	0.6710	26.6583	0.8482	28.4352	0.8966	25.9013	0.7398	30.0441	0.8702	22.9288	0.6814

**Table 6:** Performance comparison between the REMA (Rich Structure), ResNeXt, and Inception versions of REMA. ResNeXt_MS represents the ResNeXt version of REMA with multi-scale and multi-level feature fusion. SF denotes the scale factor. The numerical comparisons are accurate to 12 decimal places. The best two results are highlighted in red and blue.

MODEL	SF	BSDS100		General100		Historical		Manga109		PIRM		SET14		SET5		Urban100	
		PSNR	SSIM	PSNR	SSIM	PSNR	SSIM	PSNR	SSIM	PSNR	SSIM	PSNR	SSIM	PSNR	SSIM	PSNR	SSIM
Inception [37]	2×	29.91	0.8879	35.14	0.9510	27.14	0.8975	34.59	0.9631	33.19	0.9665	30.93	0.8922	35.92	0.9479	28.46	0.8848
ResNeXt_MS	2×	29.93	0.8883	35.17	0.9511	27.19	0.8981	34.55	0.9630	33.26	0.9668	30.91	0.8924	35.97	0.9481	28.51	0.8854
ResNeXt [38]	2×	29.91	0.8879	35.17	0.9510	27.13	0.8974	34.59	0.9630	33.24	0.9668	30.92	0.8920	35.98	0.9481	28.49	0.8848
**RichStructure**	2×	29.94	0.8887	35.16	0.9511	27.25	0.8994	34.66	0.9632	33.27	0.9668	30.95	0.8928	35.97	0.9481	28.51	0.8860
Inception [37]	4×	25.26	0.7024	28.64	0.8298	22.04	0.6722	26.64	0.8478	28.42	0.8964	25.93	0.7411	29.97	0.8693	22.90	0.6804
ResNeXt_MS	4×	25.25	0.7015	28.64	0.8298	22.02	0.6701	26.61	0.8472	28.40	0.8961	25.92	0.7402	29.98	0.8697	22.88	0.6796
ResNeXt [38]	4×	25.25	0.7018	28.63	0.8293	22.01	0.6700	26.61	0.8471	28.38	0.8958	25.91	0.7404	29.97	0.8692	22.92	0.6798
**Rich Structure**	4×	25.27	0.7026	28.67	0.8302	22.05	0.6722	26.65	0.8484	28.41	0.8962	25.97	0.7410	30.04	0.8700	22.92	0.6816
Inception [37]	8×	22.37	0.5418	24.05	0.6661	19.24	0.4494	21.10	0.6561	25.15	0.8087	21.96	0.5641	24.84	0.7190	19.55	0.4710
ResNeXt_MS	8×	22.40	0.5417	24.03	0.6649	19.24	0.4489	21.06	0.6526	25.03	0.8051	21.99	0.5645	24.79	0.7161	19.59	0.4698
ResNeXt [38]	8×	22.34	0.5409	23.99	0.6625	19.24	0.4488	20.95	0.6492	25.00	0.7964	21.89	0.5619	24.74	0.7154	19.53	0.4689
**Rich Structure**	8×	22.40	0.5430	24.10	0.6675	19.24	0.4518	21.17	0.6580	24.99	0.8065	22.01	0.5663	24.83	0.7193	19.61	0.4735

**Table 7:** REMA in the reconstruction layer. SF denotes the scale factor. #C_in denotes the input tensor’s channel count. RB w/ REMA denotes the reconstruction block with REMA. And RB w/o REMA denotes the reconstruction block without REMA. The numerical comparisons are accurate to 12 decimal places. The best result is highlighted in red.

MODEL	SF	#C_in	BSDS100		General100		Historical		Manga109		PIRM		SET14		SET5		Urban100	
			PSNR	SSIM	PSNR	SSIM	PSNR	SSIM	PSNR	SSIM	PSNR	SSIM	PSNR	SSIM	PSNR	SSIM	PSNR	SSIM
RB w/o REMA	4×	40	25.23	0.7019	28.54	0.8285	22.00	0.6707	26.53	0.8457	28.22	0.8930	25.88	0.7394	30.01	0.8689	22.86	0.6775
RB w/ REMA	4×	40	25.21	0.7014	28.55	0.8285	22.00	0.6709	26.53	0.8464	28.34	0.8946	25.86	0.7389	29.94	0.8684	22.81	0.6764
RB w/o REMA	4×	64	25.27	0.7026	28.67	0.8302	22.05	0.6722	26.65	0.8484	28.41	0.8962	25.97	0.7410	30.04	0.8700	22.92	0.6816
RB w/ REMA	4×	64	25.25	0.7015	28.59	0.8288	22.06	0.6718	26.56	0.8464	28.39	0.8952	25.92	0.7400	29.96	0.8686	22.90	0.6795
RB w/o REMA	8×	40	22.35	0.5414	24.01	0.6609	19.17	0.4467	21.03	0.6502	25.05	0.7827	21.90	0.5624	24.80	0.7173	19.46	0.4660
RB w/ REMA	8×	40	22.37	0.5420	24.08	0.6659	19.22	0.4476	21.13	0.6565	25.09	0.8052	21.94	0.5620	24.92	0.7184	19.57	0.4714
RB w/o REMA	8×	64	22.40	0.5447	24.09	0.6681	19.21	0.4498	21.17	0.6604	24.98	0.8054	22.04	0.5694	24.77	0.7209	19.61	0.4752
RB w/ REMA	8×	64	22.40	0.5430	24.10	0.6675	19.24	0.4518	21.17	0.6580	24.99	0.8065	22.01	0.5663	24.83	0.7193	19.61	0.4735

**Table 8:** Comparison with other attention modules in UNet-SR under 4×. The numerical comparisons are accurate to 12 decimal places. The best two results are highlighted in red and blue.

MODEL	BSDS100		General100		Historical		Manga109		PIRM		SET14		SET5		Urban100	
	PSNR	SSIM	PSNR	SSIM	PSNR	SSIM	PSNR	SSIM	PSNR	SSIM	PSNR	SSIM	PSNR	SSIM	PSNR	SSIM
UNet-SR_CBAM	24.93	0.6963	27.83	0.8150	21.86	0.6629	25.55	0.8263	27.98	0.8632	25.46	0.7299	29.50	0.8599	22.36	0.6586
UNet-SR_SE	24.93	0.6961	27.98	0.8172	21.85	0.6631	25.61	0.8269	28.13	0.8785	25.44	0.7307	29.35	0.8574	22.38	0.6604
UNet-SR_CA	24.92	0.6957	27.83	0.8161	21.86	0.6617	25.48	0.8239	28.08	0.8789	25.41	0.7291	29.26	0.8566	22.34	0.6575
UNet-SR_BAM	24.92	0.6963	27.95	0.8171	21.85	0.6633	25.66	0.8282	28.05	0.8740	25.43	0.7301	29.28	0.8566	22.37	0.6600
**UNet-SR_REMA**	25.01	0.6994	28.16	0.8220	21.90	0.6677	25.91	0.8335	28.27	0.8866	25.56	0.7338	29.51	0.8604	22.52	0.6663

**Table 9:** Performance comparison between REMA-SRNet and other comparative methods. HR images are center-cropped and downscaled via bicubic interpolation to generate LR image pairs for training and testing, without any data augmentation. PSNR and SSIM are computed in the RGB space. #P denotes the number of parameters(m). SF denotes the scale factor. The numerical comparisons are accurate to 12 decimal places. The best two results are highlighted in red and blue.

MODEL	#P	SF	BSDS100		General100		Historical		Manga109		PIRM		SET14		SET5		Urban100	
			PSNR	SSIM	PSNR	SSIM	PSNR	SSIM	PSNR	SSIM	PSNR	SSIM	PSNR	SSIM	PSNR	SSIM	PSNR	SSIM
VDSR [5]	0.21	2×	29.55	0.8815	34.01	0.9425	26.86	0.8890	33.27	0.9547	32.60	0.9605	30.42	0.8842	35.16	0.9428	27.47	0.8672
ESPCN [25]	0.06	2×	29.40	0.8801	33.80	0.9400	26.82	0.8876	33.06	0.9529	32.43	0.9505	30.26	0.8848	35.02	0.9421	27.05	0.8585
RCAN [27]	16.21	2×	29.90	0.8880	35.08	0.9506	27.14	0.8975	34.42	0.9628	33.28	0.9662	30.87	0.8921	35.96	0.9480	28.49	0.8864
PAN [18]	0.25	2×	29.86	0.8869	35.05	0.9502	27.19	0.8977	34.55	0.9629	33.23	0.9666	30.89	0.8920	35.91	0.9477	28.29	0.8818
A2N [36]	0.99	2×	29.85	0.8868	34.99	0.9500	27.20	0.8977	34.50	0.9626	33.21	0.9664	30.88	0.8919	35.85	0.9475	28.26	0.8813
DRLN [10]	32.84	2×	29.81	0.8865	34.91	0.9495	27.21	0.8974	34.38	0.9624	33.10	0.9652	30.79	0.8908	35.84	0.9475	28.21	0.8803
ESRGCNN [29]	2.31	2×	29.70	0.8844	34.41	0.9396	27.04	0.8910	33.89	0.9582	32.86	0.9039	30.63	0.8885	35.49	0.9441	27.86	0.8746
SwinIR [16]	10.4	2×	29.92	0.8882	35.06	0.9507	27.13	0.8971	34.61	0.9634	33.22	0.9660	30.95	0.8923	36.03	0.9481	28.37	0.8844
NLSN [20]	1.63	2×	29.86	0.8873	35.05	0.9504	27.09	0.8962	34.55	0.9627	33.11	0.9658	30.85	0.8913	36.00	0.9480	28.41	0.8846
UNetSR [30]	8.1	2×	29.41	0.8831	34.03	0.9442	26.80	0.8918	33.15	0.9564	32.60	0.9635	30.42	0.8887	35.13	0.9443	27.19	0.8645
**REMA-SRNet-M**	2.2	2×	29.89	0.8878	35.09	0.9505	27.18	0.8976	34.50	0.9627	33.30	0.9667	30.85	0.8917	35.82	0.9477	28.34	0.8833
**REMA-SRNet**	5.61	2×	29.94	0.8887	35.16	0.9511	27.25	0.8994	34.66	0.9632	33.27	0.9668	30.95	0.8928	35.97	0.9481	28.51	0.8860
VDSR [5]	0.21	4×	24.93	0.6881	27.83	0.8109	21.78	0.6518	25.30	0.8135	27.99	0.8909	25.37	0.7218	29.17	0.8514	22.31	0.6503
ESPCN [25]	0.07	4×	24.86	0.6868	27.67	0.7978	21.86	0.6445	25.02	0.7940	27.67	0.8418	25.22	0.7157	29.07	0.8417	22.09	0.6354
RCAN [27]	16.35	4×	25.17	0.6999	28.41	0.8232	21.95	0.6644	26.49	0.8403	28.10	0.8889	25.75	0.7347	29.83	0.8648	22.75	0.6740
PAN [18]	0.26	4×	25.01	0.6917	28.06	0.8164	21.92	0.6584	25.65	0.8245	28.02	0.8889	25.50	0.7274	29.41	0.8573	22.46	0.6566
A2N [36]	1	4×	25.01	0.6920	28.10	0.8174	21.92	0.6594	25.69	0.8255	28.09	0.8902	25.52	0.7278	29.43	0.8577	22.48	0.6586
DRLN [10]	32.98	4×	25.15	0.6979	28.38	0.8225	21.97	0.6663	26.32	0.8390	28.22	0.8808	25.77	0.7351	29.82	0.8650	22.71	0.6704
ESRGCNN [29]	2.31	4×	25.06	0.6944	28.08	0.8046	21.92	0.6534	25.97	0.8229	28.05	0.7900	25.59	0.7283	29.48	0.8533	22.52	0.6588
SwinIR [16]	10.45	4×	25.24	0.7025	28.57	0.8282	22.04	0.6675	26.68	0.8472	28.20	0.8842	25.92	0.7402	29.98	0.8679	22.83	0.6772
NLSN [20]	1.77	4×	25.15	0.6992	28.33	0.8222	21.98	0.6666	26.33	0.8341	27.95	0.8872	25.72	0.7335	29.80	0.8623	22.61	0.6680
UNet-SR [30]	8.11	4×	24.93	0.6963	27.97	0.8164	21.86	0.6635	25.62	0.8264	28.14	0.8727	25.41	0.7295	29.36	0.8566	22.39	0.6604
**REMA-SRNet-M**	2.23	4×	25.23	0.7019	28.54	0.8285	22.00	0.6707	26.53	0.8457	28.22	0.8930	25.88	0.7394	30.01	0.8689	22.86	0.6775
**REMA-SRNet**	5.68	4×	25.27	0.7026	28.67	0.8302	22.05	0.6722	26.65	0.8484	28.41	0.8962	25.97	0.7410	30.04	0.8700	22.92	0.6816
VDSR [5]	0.21	8×	22.09	0.5230	23.42	0.6388	19.10	0.4263	20.33	0.6015	24.15	0.7841	21.62	0.5343	24.21	0.6778	19.14	0.4383
ESPCN [25]	0.11	8×	22.19	0.5271	23.47	0.6179	19.15	0.4206	20.42	0.5901	24.35	0.6753	21.65	0.5346	24.38	0.6713	19.18	0.4342
RCAN [27]	16.49	8×	22.27	0.5354	23.79	0.6435	19.12	0.4344	20.87	0.6208	24.58	0.7620	21.84	0.5483	24.63	0.6937	19.39	0.4547
PAN [18]	0.27	8×	22.35	0.5407	23.95	0.6621	19.26	0.4469	20.95	0.6481	25.03	0.8063	21.87	0.5591	24.77	0.7137	19.49	0.4661
A2N [36]	1.01	8×	22.34	0.5406	23.96	0.6622	19.25	0.4469	20.96	0.6488	25.00	0.8059	21.85	0.5576	24.77	0.7138	19.48	0.4662
DRLN [10]	33.12	8×	22.30	0.5388	23.90	0.6537	19.07	0.4393	21.02	0.6476	24.79	0.7644	21.80	0.5562	24.61	0.7095	19.44	0.4648
ESRGCNN [29]	2.31	8×	22.24	0.5318	23.79	0.6354	19.12	0.4245	20.79	0.6174	24.78	0.7155	21.81	0.5486	24.57	0.6921	19.36	0.4514
SwinIR [16]	10.68	8×	22.38	0.5417	24.15	0.6629	19.19	0.4417	21.22	0.6501	24.88	0.7854	22.01	0.5617	25.04	0.7173	19.55	0.4680
NLSN [20]	1.91	8×	22.14	0.5293	23.56	0.6293	18.94	0.4244	20.77	0.6166	24.48	0.7566	21.72	0.5421	24.45	0.6872	19.32	0.4495
UNetSR [30]	6.77	8×	22.21	0.5384	23.70	0.6590	19.19	0.4460	20.72	0.6459	24.96	0.7795	21.73	0.5570	24.57	0.7114	19.37	0.4653
**REMA-SRNet-M**	2.47	8×	22.37	0.5420	24.08	0.6659	19.22	0.4476	21.13	0.6565	25.09	0.8052	21.94	0.5620	24.92	0.7184	19.57	0.4714
**REMA-SRNet**	6.29	8×	22.40	0.5430	24.10	0.6675	19.24	0.4518	21.17	0.6580	24.99	0.8065	22.01	0.5663	24.83	0.7193	19.61	0.4735

## Data Availability

The data used in this study are publicly available.
